# [Retracted] MicroRNA-140-5p regulates the proliferation, apoptosis and inflammation of RA FLSs by repressing STAT3

**DOI:** 10.3892/etm.2026.13147

**Published:** 2026-04-07

**Authors:** Jiehua Zhu, Jianglin Wang, Jialin Huang, Wensheng Du, Yingzhong He, Hongfei Pan, Junmin Luo

Exp Ther Med 21:171, 2021; DOI: 10.3892/etm.2020.9602

Following the publication of this paper, it was drawn to the Editor's attention by a concerned reader that the data in the ‘Control’ data panel shown for the apoptosis experiments in Fig. 4 were strikingly similar to data appearing in different form in other articles written by different authors at different research institutes, a few of which had already been published prior to the submission of this paper to *Experimental and Therapeutic Medicine*. Moreover, upon performing an independent analysis of the data in this paper, it came to light that certain of the western blot data shown in Fig. 4A, 5B and 6, and flow cytometric data in Figs. 3B and 7E, had similarly appeared elsewhere in articles written by different authors at different research institutes.

In view of the fact that the abovementioned data had already apparently been published previously, the Editor of *Experimental and Therapeutic Medicine* has decided that this paper should be retracted from the Journal. The authors were asked for an explanation to account for these concerns, but the Editorial Office did not receive a reply. The Editor apologizes to the readership for any inconvenience caused.

